# Challenges and opportunities for implementing hypofractionated radiotherapy in Africa: lessons from the HypoAfrica clinical trial

**DOI:** 10.3332/ecancer.2023.1508

**Published:** 2023-02-16

**Authors:** Elizabeth Olatunji, William Swanson, Saloni Patel, Samuel Olaolu Adeneye, Funmilayo Aina-Tofolari, Stephen Avery, Jumaa Dachi Kisukari, Katy Graef, Saiful Huq, Robert Jeraj, Adedayo O Joseph, Joerg Lehmann, Heng Li, Abba Mallum, Thokozani Mkhize, Twalib Athumani Ngoma, Andrej Studen, Krishni Wijesooriya, Luca Incrocci, Wilfred Ngwa

**Affiliations:** * Co-first authors; # Co-senior authors; 1 Johns Hopkins University School of Medicine, Baltimore, Maryland, MD 21205, USA; 2 Department of Radiation Oncology, Weill Cornell Medicine, New York, NY 10065, USA; 3 NSIA-LUTH Cancer Treatment Center, Lagos University Teaching Hospital, Lagos 100254, Nigeria; 4 Department of Radiation Oncology, Perelman Center for Advanced Medicine, University of Pennsylvania, Philadelphia, PA 19104, USA; 5 Ocean Road Cancer Institute, Dar Es Salaam 3592, Tanzania; 6 BIO Ventures for Global Health, Seattle, WA 98121, USA; 7 Department of Radiation Oncology, UPMC Hillman Cancer Center, Pittsburgh, PA 15232, USA; 8 Department of Medical Physics, University of Wisconsin, Madison, WI 53705, USA; 9 University of Ljubljana, Faculty of Mathematics and Physics, Ljubljana 1000, Slovenia; 10 Department of Radiation Oncology, Calvary Mater Newcastle, Newcastle, NSW 2298, Australia; 11 Institute of Medical Physics, The University of Sydney, Sydney, NSW 2006, Australia; 12 School of Information and Physical Sciences, University of Newcastle, Newcastle, NSW, Australia; 13 Department of Radiotherapy and Oncology, University of KwaZulu-Natal, Durban 4041, South Africa; 14 Department of Oncology, Inkosi Albert Luthuli Central Hospital, Durban 4091, South Africa; 15 Department of Clinical Oncology, Muhimbili University of Health and Allied Sciences, PO box 65001, Dar es Salaam, Tanzania; 16 Jožef Stefan Institute, Ljubljana 1000, Slovenia; 17 Department of Radiation Oncology, University of Virginia School of Medicine, Charlottesville, Virginia; 18 Department of Radiotherapy, Erasmus MC, Rotterdam, Netherlands; 19 Brigham and Women’s Hospital, Dana-Farber Cancer Institute, Harvard Medical School, Boston, USA

**Keywords:** hypofractionated radiotherapy, prostate cancer, sub-Saharan Africa, radiotherapy

## Abstract

The rising cancer incidence and mortality in sub-Saharan Africa (SSA) warrants an increased focus on adopting or developing approaches that can significantly increase access to treatment in the region. One such approach recommended by the recent Lancet Oncology Commission for sub-Saharan Africa is hypofractionated radiotherapy (HFRT), which can substantially increase access to radiotherapy by reducing the overall duration of time (in days) each person spends being treated. Here we highlight challenges in adopting such an approach identified during the implementation of the HypoAfrica clinical trial. The HypoAfrica clinical trial is a longitudinal, multicentre study exploring the feasibility of applying HFRT for prostate cancer in SSA. This study has presented an opportunity for a pragmatic assessment of potential barriers and facilitators to adopting HFRT. Our results highlight three key challenges: quality assurance, study harmonisation and machine maintenance. We describe solutions employed to resolve these challenges and opportunities for longer term solutions that can facilitate scaling-up use of HFRT in SSA in clinical care and multicentre clinical trials. This report provides a valuable reference for the utilisation of radiotherapy approaches that increase access to treatment and the conduct of high-quality large-scale/multi-centre clinical trials involving radiotherapy.

**Trial registration:**

Not available yet.

## Background

Cancer is an increasing health concern in sub-Saharan Africa (SSA). In 2020, there were 801,392 new cancer cases and 520,158 cancer-related deaths in the region [1]. The International Agency for Research on Cancer predicts a doubling of this cancer incidence and mortality in 20 years, corresponding to more than 1 million cancer-related deaths in SSA in the year 2040, if appropriate measures are not taken [1, 2]. The rising incidence of cancer in SSA has been attributed to several factors including changes in lifestyle and nutrition, rising life expectancy, infection, genetics and environmental exposures [3]. Combatting cancer in SSA requires several considerations, from developing standardised cancer registries to enhanced prevention, diagnosis and treatment approaches. Currently, few countries in the SSA region have sufficient resources to treat the majority of their cancer patients prior to the advanced progression of their disease.

**Figure 1.**
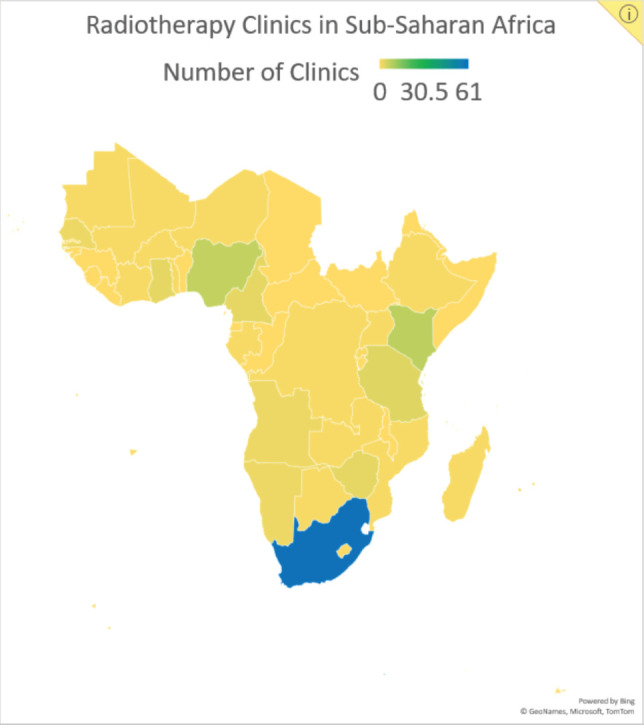
Data reported by the Directory of Radiotherapy Centers on the number of centres in SSA by country.

In addition to the limited availability of treatment resources, cancer treatment in SSA is complicated by the high costs of medical, surgical and radiation therapies, limited patient access to healthcare, shortages of skilled personnel and late patient presentation of disease [3]. Consequently, treatment strategies that prioritise both cost and resource efficiency may be practical for increasing treatment access and preventing cancer deaths. Hypofractionated radiotherapy (HFRT) is a technique with the potential to bring such cost- and resource-efficient care to SSA. In HFRT, fewer fractions of radiotherapy are delivered at larger doses than conventional radiotherapy. This technique decreases the number of required clinic visits, reducing the burden for patients, the cost of treatment and the demand on hospital staff and radiotherapy equipment [4]. These are significant benefits, especially for SSA, where radiotherapy machines and facilities are limited in number (Figure 1). Currently, there are 193 megavoltage units in SSA, with 53% of them in South Africa alone. Twenty-one of the region’s 48 countries (44%) have no megavoltage units at all [5, 6].

Existing studies have found HFRT to result in non-inferior outcomes compared to conventional radiotherapy for treating many cancers, including prostate and breast cancer, two of the most common cancers in SSA [7–11]. While existing studies highlight the benefits of HFRT, most of them were conducted in Western countries with predominantly Western populations. The under-representation of African cancer patients in existing HFRT clinical trials limits the generalisability of these studies to the SSA population. Africans have unique tumour genetics and living experiences and thus, may respond differently to treatment regimens commonly utilised in Western countries [3, 12]. HFRT clinical trials should be conducted in SSA to fill the knowledge gap on its effectiveness in this population.

**Figure 2.**
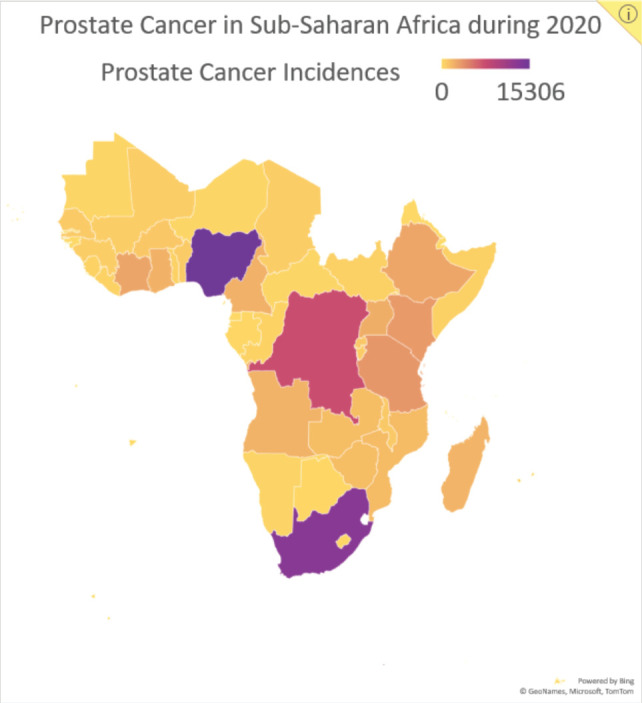
Data reported by the Global Cancer Observatory on the number of new prostate cancer incidences in SSA by country during 2020.

Considering prostate cancer has the highest cancer-related incidence (Figure 2) and mortality for men in SSA, and the potential benefits of HFRT for prostate cancer, we began HypoAfrica, a multicentre feasibility study led by SSA-based oncologists to examine the utility of HFRT for localised prostate cancer in the SSA setting. Each week, the study teams at the study’s three sites (Lagos, Nigeria; Dar es Salaam, Tanzania; and Durban, South Africa) meet with a support team of radiation oncology and other oncology professionals from the United States, Africa, Europe and Australia. These meetings allow the various sites to share updates, review study proceedings and troubleshoot problems. In addition to valuable information on the utility of HFRT in SSA populations, the HypoAfrica study also provides a crucible for identifying challenges or barriers to broader adoption of HFRT and to conducting high-quality multicentre – or otherwise large-scale – clinical trials in SSA that involve HFRT. This article outlines the challenges encountered during this study and the solutions employed to overcome these challenges. Furthermore, we discuss the opportunities that have emerged from this multicentre research collaboration, to provide a reference for future studies, and to encourage greater adoption of HFRT in SSA as recommended in the Lancet Oncology Commission for SSA [3].

## Methods

### HypoAfrica clinical trial

As described above, the aim of the HypoAfrica study, initiated in 2022, is to assess the feasibility of applying moderate HFRT for localised prostate cancer in SSA. This clinical trial was designed to mirror the European CHHiP study [8]. A total of 182 participants will be recruited across three sites – the Nigeria Sovereign Investment Authority-Lagos University Teaching Hospital Cancer Center in Lagos, Nigeria, the Muhimbili University of Health and Allied Sciences and Ocean Road Cancer Institute in Dar es Salaam, Tanzania, and the Inkosi Albert Luthuli Central Hospital in Durban, South Africa. As in the CHHiP trial, included participants are men older than 16 years with histologically confirmed localised low-intermediate-high risk prostate cancer, no active malignancy up to 5 years prior and no previous pelvic radiotherapy, radical prostatectomy or androgen suppression [8].

The patients will be treated using 3-Dimensional Conformal Radiotherapy and Intensity Modulated Radiotherapy (IMRT) techniques on linear accelerators (Nigeria: Varian Vital Beam × 2 machines; Tanzania: Varian Vital Beam × 2 machines; South Africa: Varian IX Trilogy × 2 machines). Patient-specific quality assurance (QA) will be performed for IMRT treatments using either film or portal dosimetry techniques. Image-guidance is provided by portal films or images compared to digitally-reconstructed-radiographs from the preceding CT simulation for position verification. More information on the protocol can be found here (doi: 10.6084/m9.figshare.21913200).

The study will include 1 year of patient recruitment (ongoing) and treatment, and 5 years of patient follow-up. The primary endpoints for this study are toxicity at the end of treatment and toxicity 3–24 months post-treatment. Secondary endpoints are PSA failure-free survival at 5 years, relapse-free survival at 5 years, overall survival at 5 years and cost-effectiveness of HFRT. Weekly team meetings, as described above, were organised to discuss and address opportunities and barriers to implementing HFRT in SSA settings.

### HFRT survey

Prior to the start of the HypoAfrica trial, we conducted an online survey to assess the readiness of African radiotherapy clinics to adopt curative HFRT. Criteria for readiness were based on the stereotactic body radiotherapy recommendations in the American Association of Physicists in Medicine Task Group Report 101 and other professional organisations [13, 14]. The survey was distributed to healthcare providers in radiotherapy clinics across SSA and was accessible via Google Forms for 3 months. The survey included questions about current HFRT practices and infrastructure utilised in the clinics, and the respondents’ clinical role and training level. As South Africa has substantially more radiotherapy centres than the rest of the region, the continental survey data may show a slight bias towards an overestimated ‘readiness’ for HFRT practices. Additional details about this survey can be found in Swanson *et. al* [13]. The survey results were analysed to elucidate challenges observed in implementing the HypoAfrica trial.

## Results

### Challenges identified and solutions employed

#### Quality assurance

Radiotherapy QA and treatment protocol adherence have been found to correlate positively with patient survival [15–17], highlighting the importance of QA in radiation oncology and clinical trials involving radiotherapy. QA is even more critical when administering higher intrafraction doses of radiotherapy, as in HFRT. In the HypoAfrica study context, effective QA ensures that each radiotherapy machine delivers the expected output, the radiation dose distribution delivered to each patient matches their treatment plan, each treatment planning system is modelled with expected machine characteristics, each radiotherapy machine has image-guidance where relevant, intra/inter-fraction patient set-up is reproducible and end-to-end testing with the same phantom with disease site-specific credentialing occurs. Effective QA in our multicentre trial also requires that reliable and comparable QA tools are used across the three sites.

**Figure 3.**
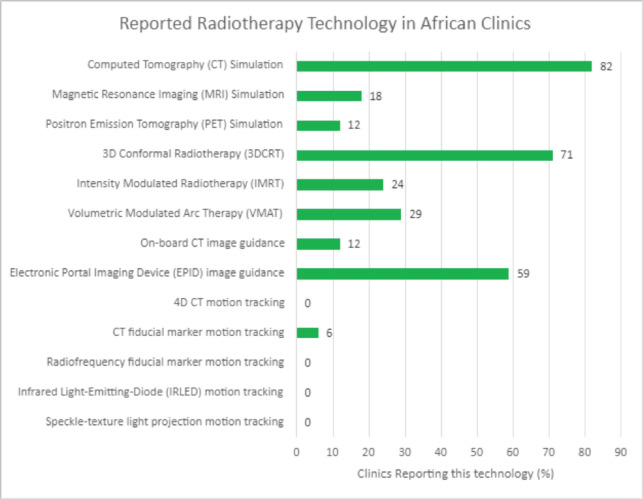
Reported accessible radiotherapy technology in African radiotherapy centres.

In assessing QA needs for implementing the HypoAfrica clinical trial, the availability of needed infrastructure to ensure high-QA was identified as a challenge: not all sites had the equipment and established routines that facilitate the required QA for implementing HFRT. This infrastructure challenge appears consistent with findings in the survey (Figure 3) and corroborates the need for considering infrastructure needs when considering adoption of HFRT in SSA. To address these needs, QA tools and their corresponding training were provided. Radiochromic film [18] and film scanners were obtained for film-based QA. Training and support were provided for using electronic portal imaging devices for QA. Furthermore, Klio (Luca Medical Systems) [19], a cloud-based solution that allows managing and tracking extensive QA data to monitor trends in the output of the megavoltage units, was introduced. Another example of fulfilling the infrastructure needs at our sites was the introduction of VESPA [20, 21], a remote audit that allows for external peer review of dosing and comparison of dosimetric parameters and variations between the centres. This latter solution also availed efforts to harmonise QA across the different sites.

#### Machine maintenance challenge

Another challenge identified during the HypoAfrica trial was machine maintenance, an issue highlighted in previous studies on radiotherapy in SSA [22–24]. Machine breakdown can delay treatment and clinical trial progress and increase patient wait time, resulting in disease advancement, treatment abandonment and other issues. Our sites observed several instances of linear accelerator breakdown, mainly tied to power surges, chiller breakdown and overuse of limited machines. To minimise machine downtime, sites were encouraged to practice preventive maintenance, namely employing uninterruptible power supplies (UPS) and checking the connections of the UPS to the linear accelerators, utilising surge protectors, having stable service contacts with reliable vendors, monitoring the humidity where the linear accelerators are located and proactively replacing parts by anticipating breakdown before it occurs (for example, by monitoring multileaf collimator performance with log files daily). In addition to preventive maintenance, it is important that cancer treatment facilities have experienced engineers who can diagnose and fix faulty hardware. The lack of such staff at the study sites has occasionally led to some treatment delays. In the future, increased training opportunities for the on-site machine engineers may help to prevent complications from machine breakdown.

#### Data and quality harmonisation

While multicentre clinical trials have many advantages, including increased sample size, greater generalisability and the ability to conduct more robust analyses, the challenge of harmonising study components across all sites comes with them. Indeed, homogenising patient recruitment, data collection and nomenclature to ensure the data collected are comparable and protocol-adherent are all critical to the success of a multicentre clinical trial. Harmonisation of QA techniques in the HypoAfrica study has been described in the previous section. For patient recruitment, the inclusion criteria outlined in the study protocol facilitated the selection of patients at each study site that matched the target population. To homogenise data collection, we introduced a centralised platform with data collected in a relational database within the existing Networks of Imaging eXcellence (NIX) infrastructure, used in other multi-centre studies [25]. NIX allows homogenisation of the processes for acquisition and collation of patient imaging and other clinical data and provides a legal, ethical and logistic framework for linking geographically, politically and functionally disparate data sets with each other, and provides an environment for cross-validation of derived biomarkers and models. The clinical information was captured via data collection units and digitised clinical report forms that were presented to clinicians for targeted collection of data. We then employed a digital platform, LabKey Server (Labkey) [26], that auto-populates the collected data into a centralised database, facilitating the integration, sharing and analysis of our entire dataset. This standardised data collection and entry strengthens the data’s integrity and reduces the time spent interpreting and analysing data.

#### Opportunities for facilitating broader adoption of HFRT in SSA

##### Continuous education

**Figure 4.**
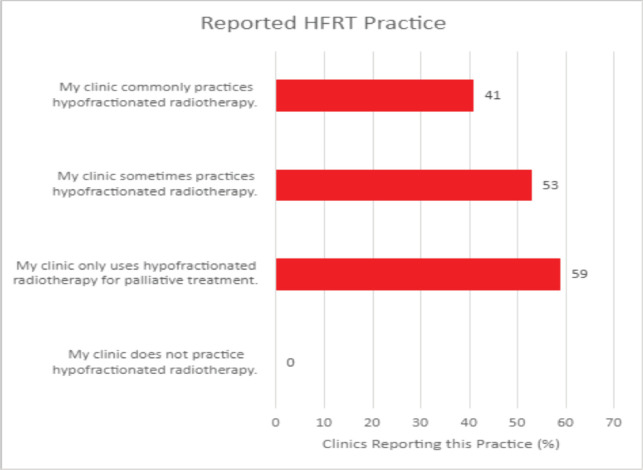
Reported HFRT familiarity among African radiotherapy centres.

**Figure 5.**
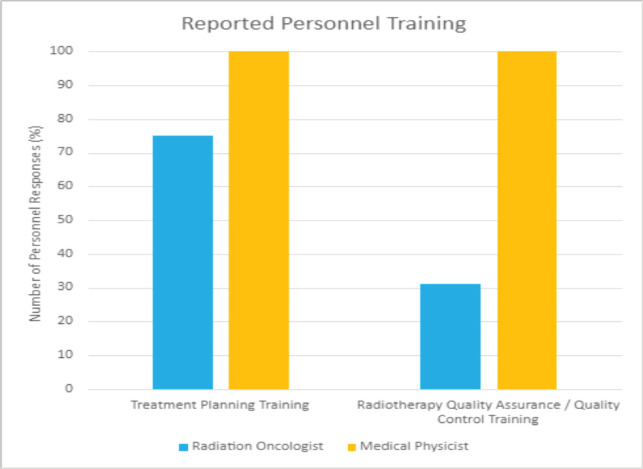
Reported training received by radiation oncologists and medical physicists in African radiotherapy centres.

In conducting the HypoAfrica trial, the need for continuous medical education (CME; a hallmark of career development beneficial for ensuring oncology health professionals continue to grow their capacity as new evidence-based approaches emerge) became apparent. CME would facilitate greater adoption of approaches like high-quality HFRT in SSA, and is currently utilised for this function in the United States. Our survey anticipated that minimal additional training for practicing HFRT was necessary for many clinics, since 59% of survey respondents reported that their site already practises HFRT for palliative care (Figure 4). However, Figure 5 from the survey provides additional perspective on SSA site training levels, indicating that oncologists are less familiar with QA protocol and treatment planning techniques. While these findings include sites not involved in the HypoAfrica study, they provide valuable insight into the readiness of oncology professionals in SSA for broader adoption of HFRT. These findings also encouraged us to employ our existing collaboration to develop high-quality HFRT continuous education programmes delivered via the Global Oncology University training platform [27] organised by Radiation Knowledge [28], the Global Health Catalyst [29] and BIO Ventures for Global Health [30]. These training programmes will advance the uptake of high-quality curative HFRT in SSA.

##### Imaging and Radiation Oncology Core in Africa

Besides creating a platform for continuous education regarding HFRT, the HypoAfrica study has highlighted other opportunities that can facilitate greater adoption or scale-up of HFRT in clinical practice and trials in SSA. One opportunity that has emerged as a longer-term solution for some of the challenges seen in the HypoAfrica trial is the development of a consortium with funding proposal towards establishing an Imaging and Radiation Oncology Core (IROC) in Africa. In the United States, IROC supports multi-centre clinical trials by providing radiotherapy quality control programmes and equipment to ensure that the radiation doses administered to patients in trials are ‘accurate and comparable between participating institutions’ [31]. IROC Africa will offer an Afrocentric solution to help ensure that African research institutions are credentialed before participating in clinical trials, and that they have the appropriate QA resources and human capacity to carry out their trials confidently and successfully. Establishing an IROC Africa would also encourage more oncology centres in SSA to participate in clinical trials, increase data quality across the region and promote standardisation of treatment and treatment quality. This increased amount of high-quality radiotherapy trials would grant patients in SSA better access to quality care and the latest treatment techniques.

##### Telehealth

A third opportunity we identified through HypoAfrica to facilitate wider adoption of HFRT in SSA is telehealth. This study has highlighted an opportunity and model for global health in radiation oncology leveraging advanced information and communication technologies to provide remote QA, peer review and support through teleoncology. HypoAfrica has also provided significant opportunities for Western oncology health professionals and trainees to participate in global health experiences, working across cultures and learning about the remarkable work done by healthcare professionals in resource-limited settings and different cultures. Collaboration through the HypoAfrica project has indubitably tested a model for telehealth that can be scaled in North-South collaborations.

## Discussion

The HypoAfrica study emerged out of the desire to improve access to cancer treatment in SSA by introducing therapies that are resource-efficient and equivalent to conventional therapies. HFRT is a modern technique that guarantees such efficiency and results in non-inferior outcomes compared to conventional radiotherapy in studies with predominantly Western populations. The unique tumour genetics and living experiences of the SSA population warrant trial assessment of the effectiveness and feasibility of HFRT in SSA. Our multicentre feasibility study examines the feasibility of applying HFRT for localised prostate cancer in the SSA setting. This article details the challenges we encountered while implementing the HypoAfrica study, as well as the opportunities that emerged. Notably, we encountered challenges to QA, study harmonisation, and machine maintenance and noted opportunities for continuous education, IROC Africa and telehealth. Combatting the challenges was essential to maintain the integrity of the trial and to ensure patient safety. The software introduced as solutions (i.e. Klio, VESPA, NIX, Labkey) were well received by each site, but required that the on-site researchers receive additional training to appropriately utilise them. Based on a review of the literature, this is one of the first multi-centre clinical trials involving radiotherapy in SSA. We hope that the lessons from our trial will serve as valuable recommendations for future oncology clinical trials in SSA, including planned trials combining HFRT with immunotherapy.

At present, the strengths of our study include: 1) the creation of a framework for radiotherapy QA at three sites; 2) multicentre study harmonisation that can be used in future clinical trials in SSA; 3) the establishment of a multi-continent collaboration between oncology health professionals; 4) increased awareness about barriers to cancer treatment and the implementation of clinical trials in SSA; 5) potential for improved outcomes for patients enrolled in our study; 6) implementation of existing software (Labkey, Klio and VESPA) at clinical sites in SSA and 7) opportunities to improve these tools based on this experience.

Limitations of our study include its confinement to three hospitals in three SSA countries. However, the additional perspective provided by the survey suggests that some of the challenges apply across many SSA countries. The HypoAfrica trial is only focused on prostate cancer, and the challenges observed may somewhat differ when HFRT is implemented for other cancer sites, like breast cancer where evidence-based HFRT has also been established. More studies involving more sites are needed to pellucidly investigate the barriers and facilitators for implementing HFRT in clinical practice in SSA. Such investigations would further advance our understanding of the utility and practicality of implementing HFRT in the SSA setting.

## Conclusion

Through the HypoAfrica clinical trial, we identified potential barriers to the broader adoption of HFRT in SSA. Opportunities identified could be leveraged to strengthen the capacity of SSA countries to scale up HFRT and significantly increase access to treatment. We hope that the lessons from our experience will serve as valuable resources for subsequent studies. This project provides a significant impetus for broader implementation of scientific research assessing the barriers and facilitators of adopting evidence-based approaches like HFRT and telehealth to increase access to care in SSA.

## Conflicts of interest

The authors declare that they have no conflicts of interest.
